# Effect of estimated plasma volume status and left atrial diameter on prognosis of patients with acute heart failure

**DOI:** 10.3389/fcvm.2023.1069864

**Published:** 2023-01-25

**Authors:** Xiaomin Chen, Guoli Lin, Caizhi Dai, Kaizu Xu

**Affiliations:** Department of Cardiology, The Affiliated Hospital of Putian University, Putian University, Putian, China

**Keywords:** estimated plasma volume status, left atrial diameter, acute heart failure, hematocrit, hemoglobin

## Abstract

**Objective:**

Acute heart failure (AHF) is a frequent cardiovascular emergency presenting with high mortality as well as readmission rates. The aim was to investigate the predictive value of estimated plasma volume status (ePVs) and left atrial diameter (LAD) for the prognosis of patients with AHF.

**Methods:**

Clinical profiles were collected from 259 cases of AHF patients at the Affiliated Hospital of Putian University between September 2019 and October 2021.

**Results:**

Six patients lost follow-up, resulting in 253 patients enrolled. Cardiogenic death and heart failure readmission during follow-up were defined as major cardiovascular events (MACE) group, other patients were defined as Non-MACE group. Apart from age, no significant differences were found between the two groups in demographics and comorbidities. The comparison between the two groups was statistically significant in terms of ePVs, LAD, and N-terminal-pro B-type natriuretic peptide (Nt-pro-BNP). On binary logistic regression analysis, ePVs (OR = 2.061, 95% CI 1.322∼3.214, *P* = 0.001), LAD (OR = 1.054, 95% CI 1.012∼1.098, *P* = 0.011), and Nt-pro-bnp (OR = 1.006, 95% CI 1.003∼1.010, *P* = 0.036) as predicting factors for MACE. Kaplan-Meier analysis indicated that the risk for cardiogenic death increasing with ePVs (*p* < 0.05).

**Conclusion:**

Estimated plasma volume status and LADs have some predictive value in assessing cardiogenic death and heart failure readmission in patients with AHF.

## Introduction

Heart failure is a life-threatening and costly condition caused by structural and functional irregularities within the heart. It results in systolic and diastolic dysfunction, failure of cardiac output to meet the metabolic needs of the body, and inadequate perfusion of organs and tissues, leading to a series of clinical syndromes (1). Age-adjusted rates of heart failure may be declining in developed countries, possibly reflecting better management of cardiovascular disease, but overall rates are increasing due to aging. Currently, the prevalence of heart failure in Chinese residents aged 35 years or older is 1.3%, with approximately 13.7 million patients with heart failure (2). Acute heart failure is a symptom and sign that appears or worsens rapidly following abnormal cardiac function and is related to an increased in plasma natriuretic peptide levels.

Hematocrit is the proportion of erythrocytes to the whole blood volume and can reflect the severity of anemia and blood volume load. Anemia leads to myocardial ischemia and hypoxia, which results in compensatory increases in stroke volume and heart rate, secondary to myocardial remodeling, cardiac structural changes, and reduced cardiac systolic and diastolic functions (3). In aged patients who had preserved ejection fraction as well as mild reduced heart failure, decreased hematocrit was a risk factor for major adverse cardiovascular events in the vulnerable period. The lower the hematocrit, the worse the prognosis, the higher the readmission rate and mortality (4).

Plasma volume (PV) was a marker of volume overload based on the Duarte formula estimated on the basis of erythrocyte volume and hemoglobin (5). Estimated PV status (ePVs) was the use of hematocrit and hemoglobin from Strauss derivative Duarte calculated in the formula, specific as shown below: ePVs (mL/g) = 100 × (1–hematocrit)/hemoglobin (g/dL) (6). It was correlated with clinical outcomes in patients suffering from heart failure with reduced ejection fraction and in patients with acute heart failure. The higher the plasma volume status, the worse the prognosis (7).

The left atrium is an important factor in maintaining left ventricular filling, which has reserve function, pipeline function and pump function. Atrial function was closely related to the occurrence of heart failure. Left atrial dysfunction could be used as an independent predictor of the prognosis of heart failure (8). Left atrial enlargement indicated poor prognosis, and left atrial reduction after treatment could be used as a prognostic indicator (9).

Acute heart failure (AHF) is a common critical disease in the Department of cardiovascular medicine, with high mortality and readmission rate. Currently, there are few reports on the relationship between estimated plasma volume status and left atrial diameter on the prognosis of patients with acute heart failure. We have analyzed the factors influencing cardiovascular events in patients with acute heart failure in order to enhance the management of patients with heart failure and reduce readmission and mortality rates in patients with acute heart failure.

## Materials and methods

### Study populations

The study conformed to the declaration of Helsinki, and obtained the Affiliated Hospital of Putian University ethics review board for approval.

It was a retrospective study and collected data on the Hospital Digital Information System. Such data did not involve identifiable personal data; therefore, informed consent was not required to approve our study.

A total of 259 patients with acute heart failure presented at The Affiliated Hospital of Putian University from September 2019 to October 2021 were enrolled. Acute heart failure diagnosis in line with Universal definition and classification of heart failure published in the European Journal of Heart Failure in 2021 (10). Exclusion criteria: active bleeding and transfusion; new onset or previous history of atrial fibrillation/flutter; moderate to severe mitral valve disease; advanced renal disease requiring chronic renal replacement therapy; cerebral hemorrhage and other severe cerebrovascular disease; severe hepatic insufficiency; malignancy. We followed up all patients for 1 year. For the follow-up period, cardiogenic death and readmission due to heart failure were defined as the MACE group, and non-cardiogenic death and readmission not due to heart failure were defined as the non-MACE group.

### Data collection

Demographic, clinical parameters and laboratory test results were assembled. All data collected through medical records.

### Laboratory indicators

Blood samples were drawn on admission by the nurse in the vacuum vessel treated with ethylene diamine tetra-acetic acid. The automated hematology analyzer (CAL8000; Mindray, Shenzhen, China) measured parameters such as hematocrit and hemoglobin using the electrical impedance method for complete blood count. ePVs was derived by 100 × (1–hematocrit)/hemoglobin in g/dL. Automatic fluorescence immunoanalyzer (Getein1600; Geteinbiotech, Nanjing, China) measured N-terminal B-type natriuretic peptide (NT-pro-BNP) by dry immunofluorescence method.

### Echocardiography

For all patients, measurements were taken using a GE Vivid 7 echocardiography device (General Electric, Boston, MA, USA). Measured the left atrial diameter (LAD) under the long axis of the parasternal left ventricle. Measurement of left ventricular ejection fraction (LVEF) by Simpson’s method under apical four-chamber and two-chamber views.

### End-point

After discharge patients were followed up at 1, 2, 3, 6, and 12 months. Follow-up methods included outpatient follow-up, phone calls, WeChat, etc. The incidence of end-point events were recorded and follow-up was terminated after cardiogenic death. End-point events were defined as major cardiovascular events (MACE) during the follow-up period, including cardiogenic death and readmission for heart failure.

### Statistical analysis

The statistical package for social sciences software (SPSS 22.0 for Windows, IBM, Armonk, NY, USA) was used for all statistical analyses. The continuous variables were shown as mean ± standard deviation and were tested by independent samples *t*-test for comparison. The categorical variables were shown as proportions, and the chi-square test was used for differences between categorical variables. All factors at entry that were statistically significant between groups with a *p*-value <0.05 and variables considered of relevant clinical interest were included in the logistic regression to identify the independent predictors of the endpoint event. Receiver operating characteristics (ROC) curve assess the predictive value of left atrial diameter and ePVs for MACE. Kaplan-Meier survival curves were used for survival analysis and log-rank tests were used for comparison of differences. The difference was considered statistically significant at a *P*-value < 0.05 (two-tailed test).

## Results

### Demographic characteristics

Six patients were lost to follow-up, and 253 patients were finally enrolled. There were 75 cases in the MACE group, of which 44 were cardiogenic deaths. There were 178 in the Non-MACE group. In the MACE group, 46 were males and 29 were females, average age was (68.15 ± 11.65), comprising 18 patients with coronary artery disease, 24 patients with hypertension and 20 patients with diabetes. In the non-MACE group, 120 were males and 58 were females, average age was (62.03 ± 11.70), comprising 40 patients with coronary artery disease, 55 patients with hypertension and 38 patients with diabetes mellitus. No statistical significance was found between the two groups in terms of gender, smoking, body mass index and comorbidities (*p* > 0.05). The age and rate of lower limb edema of MACE group was higher than that of the non-MACE group. The values were detailed in Table 1.

**TABLE 1:** Clinical and demographic properties of two groups.

Variable	MACE group (*n* = 75)	Non-MACE group (*n* = 178)	*P*-value
Age (years)	68.15 ± 11.65	62.03 ± 11.70	0.001
Male (%)	46 (61.33)	120 (67.41)	0.386
Coronary artery disease (%)	18 (24.00)	40 (22.47)	0.870
Hypertension (%)	24 (32.00)	55 (30.89)	0.883
Diabetes (%)	20 (26.67)	38 (21.35)	0.413
Smoke (%)	18 (24.00)	38 (21.35)	0.870
Body mass index (kg/m^2^)	23.98 ± 2.61	24.34 ± 3.16	0.392
Lower limb edema (%)	63 (84)	117 (65.73)	0.004
**Medication at discharge, *N* (%)**			
ACEi/ARB/ARNI	58 (77.33)	125 (70.22)	0.283
Loop diuretics	65 (86.66)	134 (75.28)	0.045
Spironolactone	66 (88)	131 (73.59)	0.013
Beta-blocker	66 (88)	122 (68.54)	0.001

ACEi, angiotensin-converting enzyme inhibitor; ARB, angiotensin receptor blocker; ARNI, angiotensin receptor neprilysin inhibitor.

### Baseline characteristics of patients

Hemoglobin, hematocrit, red cell distribution width (RDW), ePVs, blood urea nitrogen (BUN), Sodium, albumin, C-reactive protein (CRP), D-dimer, Nt-pro-bnp, LVEF, and LAD were all significantly different between the two groups. White-blood cells (WBC), total cholesterol (TC), low-density lipoprotein cholesterol (LDL), creatinine, high-density lipoprotein cholesterol (HDL), BUN/creatinine, uric acid, triglycerides (TG), potassium, and hemoglobin A1c were not significantly different between the two groups. The values were detailed in Table 2.

**TABLE 2:** Baseline characteristics of the two groups.

Variable	MACE group (*n* = 75)	Non-MACE group (*n* = 178)	*P*-value
ePVs	5.26 ± 1.73	4.20 ± 0.80	0.001
WBC count (10^9^/L)	12.27 ± 7.09	10.90 ± 3.95	0.051
Hemoglobin (g/L)	125.71 ± 21.14	141.92 ± 15.77	0.001
Hematocrit (%)	37.16 ± 6.22	41.55 ± 4.27	0.001
RDW-SD (%)	43.21 ± 4.18	41.55 ± 3.09	0.002
Albumin (g/L)	38.15 ± 4.48	40.91 ± 3.58	0.001
TG (mmol/L)	1.38 ± 1.75	1.74 ± 2.07	0.183
TC (mmol/L)	4.42 ± 1.35	4.78 ± 1.36	0.053
HDL (mmol/L)	1.04 ± 0.29	1.05 ± 0.25	0.909
LDL (mmol/L)	3.21 ± 0.92	3.27 ± 1.23	0.720
BUN (mmol/L)	6.20 ± 3.45	5.37 ± 2.17	0.022
Creatinine (umol/L)	83.67 ± 35.40	78.441 ± 22.71	0.162
BUN/creatinine	18.79 ± 8.45	17.19 ± 5.38	0.073
Uric acid (umol/L)	393.89 ± 127.97	387.43 ± 104.31	0.945
Potassium (mmol/L)	3.90 ± 0.80	3.82 ± 0.45	0.312
Sodium (mmol/L)	136.44 ± 4.12	137.83 ± 3.45	0.011
D-dimer (ug/mL)	1.76 ± 4.94	0.65 ± 1.13	0.008
HbA1c (%)	7.07 ± 2.01	6.97 ± 1.69	0.098
CRP (μg/L)	18.77 ± 29.08	10.54 ± 21.21	0.015
Nt-pro-bnp (Pg/ml)	4973.832 ± 7587.05	2084.01 ± 4096.35	0.002
**Echocardiography**			
LAD (mm)	43.23 ± 9.37	38.11 ± 8.07	0.001
E/e’ ratio	16.01 ± 4.81	14.09 ± 5.15	0.006
LVEF (%)	53.21 ± 8.89	57.992 ± 8.18	0.002
HFpEF (*N*, %)	51 (68%)	152 (85.39%)	0.003
HFmrEF (*N*, %)	19 (25.33%)	18 (10.12%)	0.003
HFrEF (*N*, %)	5 (6.67%)	8 (4.49%)	0.536

BUN, blood urea nitrogen; CRP, C-reactive protein; ePVs, estimated plasma volume status; HbA1c, hemoglobin A1c; HDL-C, high-density lipoprotein cholesterol; HFmrEF, heart failure with mid-range ejection fraction; HFrEF, heart failure with reduced ejection fraction; HFpEF, heart failure with preserved ejection fraction; LAD, left atrial diameter; LDL-C, low-density lipoprotein cholesterol; LVEF, left ventricular ejection fraction; Nt-pro-BNP, N terminal pro B type natriuretic peptide; RDW, red cell distribution width; TC, total cholesterol; TG, triglyceride; WBC, white blood cell.

### Predictive indicators for MACE.

In correlation analysis, the ePVs was correlated with Nt-pro-bnp (*r* = 0.122, *p* < 0.05), LAD (*r* = 0.168, *p* < 0.05), E/e’ ratio (*r* = 0.253, *p* < 0.05). The Nt-pro-bnp was correlated with LAD (*r* = 0.159, *p* < 0.05). The LAD was correlated with E/e’ ratio (*r* = 0.137, *p* < 0.05). The values were detailed in Table 3.

**TABLE 3:** Correlation of indicators.

Variables	ePVs		Nt-pro-bnp		LAD		E/e’ ratio	
	***r***	***P***	***r***	***P***	***r***	***P***	***r***	***P***
ePVs	1	–	0.122	0.026	0.168	0.040	0.253	0.001
Nt-pro-bnp	0.122	0.026	1	–	0.159	0.006	0.055	0.193
LAD	0.168	0.040	0.159	0.006	1	–	0.137	0.015
E/e’ ratio	0.253	0.001	0.055	0.193	0.137	0.015	1	–

ePVs, estimated plasma volume status; LAD, left atrial diameter; Nt-pro-BNP, N terminal pro B type natriuretic peptide.

Analysis of binary logistic regression indicated that ePVs (OR = 2.061, 95% CI 1.322∼3.214, *P* = 0.001), LAD (OR = 1.054, 95% CI 1.012∼1.098, *P* = 0.011) and Nt-pro-bnp (OR = 1.006, 95% CI 1.003∼1.010, *P* = 0.036) were MACE predictors in acute heart failure patients. The values were detailed in Table 4.

**TABLE 4:** Logistic regression analysis for major cardiovascular events (MACE).

Variable	β	Wals	*P*	OR	95% CI
ePVs	0.723	10.180	0.001	2.061	1.322∼3.214
LAD	0.053	6.471	0.011	1.054	1.012∼1.098
RDW	0.091	2.501	0.114	1.095	0.978∼1.226
Albumin	-0.087	2.057	0.151	0.916	0.813∼1.032
BUN	-0.079	0.863	0.353	0.924	0.782∼1.092
Sodium	-0.023	0.170	0.680	0.977	0.875∼1.091
D-dimer	0.192	2.271	0.132	1.211	0.944∼1.554
CRP	0.001	0.001	0.983	1.007	0.985∼1.016
Nt-pro-bnp	0.001	4.394	0.036	1.006	1.003∼1.010
Age	0.028	2.825	0.093	1.029	0.995∼1.064
LVEF	-0.087	14.665	0.001	0.917	0.877∼0.958

BUN, blood urea nitrogen; CRP, C-reactive protein; ePVs, estimated plasma volume status; LAD, left atrial diameter; LVEF, left ventricular ejection fraction; Nt-pro-BNP, N terminal pro B type natriuretic peptide; RDW, red cell distribution width.

### Receiver operating characteristic curve

**FIGURE 1:**
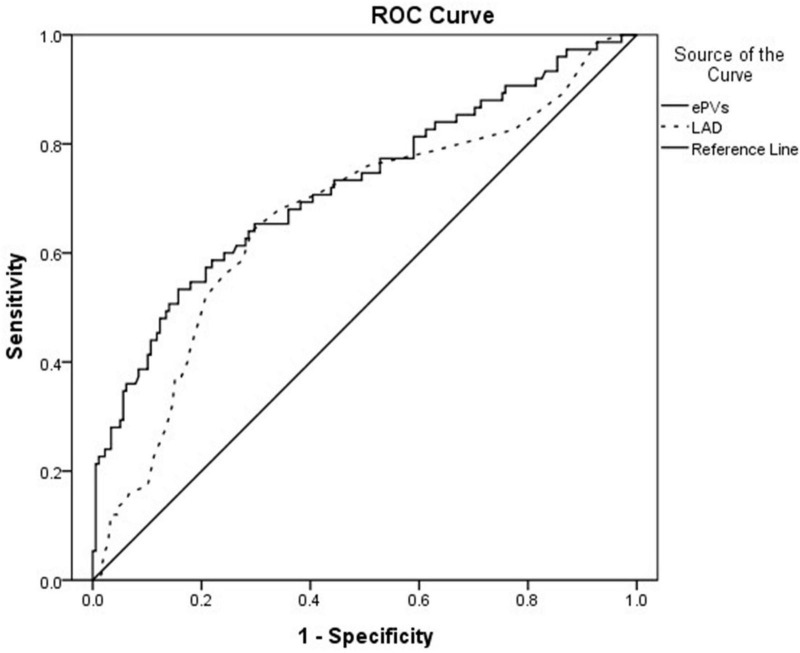
Receiver operating curve showing the area under the curve (AUC) for estimated plasma volume status (ePVs) and left atrial diameter (LAD).

The ePVs area under the curve was 0.721 (95% CI: 0.648∼0.794, *p* < 0.001) with an optimal cut point value of 4.98 for predicting MACE, 64% sensitivity and 71.3% specificity; The LAD area under the curve was 0.668 (95% CI: 0.592∼0.745, *p* < 0.001) with an optimal cut point value of 38.5 mm for predicting MACE, 64% sensitivity and 70.8% specificity (Figure 1).

### The Kaplan–Meier survival analysis for cardiogenic death

**FIGURE 2:**
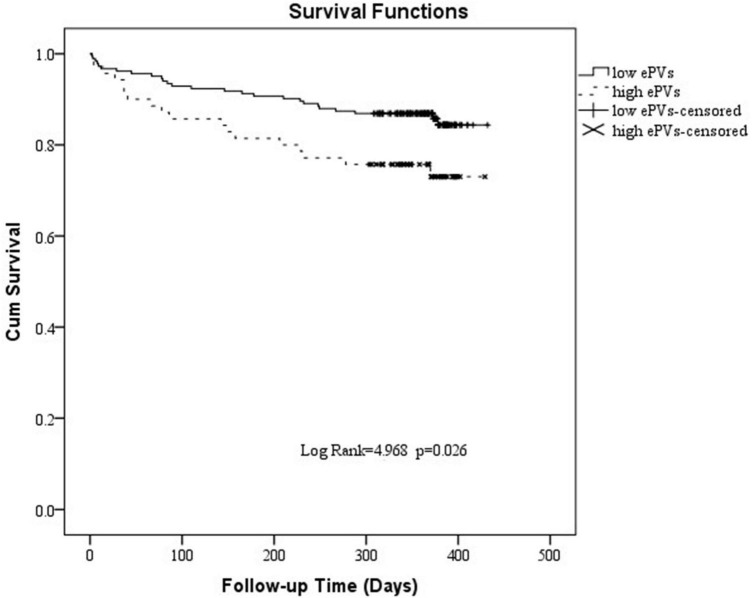
Kaplan-Meier survival curve of 1 year cardiogenic death in patients with high estimated plasma volume status (ePVs) and low ePVs groups.

Analysis of the ROC curve indicates an optimal ePVs threshold of 4.98 for MACE. Patients were divided into a high ePVs (ePVs ≥ 4.98) group (*n* = 70) and a low ePVs (ePVs < 4.98) group (*n* = 183). At 1 year follow-up, cardiac mortality was higher in the group with high ePVs than in the group with low ePVs (*P* = 0.027) (Table 5). Kaplan survival Meier analysis indicates a similar tendency (Figure 2).

**TABLE 5:** One-year follow-up results of patients in the high and low estimated plasma volume status (ePVs) groups.

	Cardiogenic death group	Survival group
High ePVs (*n* = 70)	18 (25.71)	52 (74.29)
Low ePVs (*n* = 183)	26 (14.21)	157 (85.79)
X^2^	–	4.666
*P*-value	–	0.027

ePVs, estimated plasma volume status.

## Discussion

This single-center retrospective study evaluated the predictive value of ePVs and LAD in patients with AHF. The study showed that ePVs were higher in MACE group than in controls, and the LAD was larger than in controls. ePVs, LAD, and Nt-pro-bnp were MACE predictors in acute heart failure patients. ROC analysis showed that the optimal ePVs threshold for MACE was 4.98 with sensitivity of 64%, and specificity of 71.3%. At 1 year follow-up, cardiac mortality was higher in the group with high ePVs than in the group with low ePVs.

Acute heart failure is defined as signs and/or symptoms of rapid or progressive heart failure that are severe enough to warrant emergency medical attention. Patient with AHF required urgent assessment and initiation or intensification of treatment (11). AHF is the primary cause of hospitalization for patients aged >65 year and is related to high rates of mortality and readmission. Mortality rates in hospital range between 4 and 10% (12). One year after discharge, the mortality rate could be 25–30%, and the mortality or readmission rate could be more than 45% (13). In our study, the incidence of MACE events at 1 year follow-up was 29.64%. This may be related to race and region. We also found that the incidence of MACE events within 30 days was approximately 4% and the incidence of MACE events within 180 days was approximately 17%. The incidence of MACE events may be higher if the duration of follow-up is extended. And the study population was mainly composed of HFpEF or HFmrEF patients, only about 5% of patients with HFrEF. This may also be the reason for the low incidence of MACE.

Congestion was the main cause of hospitalization for acute decompensated heart failure and was associated with a poor prognosis (14). It was essential to assess the intensity of congestion in order to obtain the best possible treatment for heart failure. Plasma volume, which is the intravascular portion of the extracellular fluid volume, could be gauged with radio-labeled tracer molecules by standard dilution techniques (15). Non-invasive PV evaluation was essential for the treatment of heart failure patients, but was not possible due to the unreliability of clinical signs and symptoms (16). A correlation had now been demonstrated between PV estimated from hemoglobin/hematocrit and PV estimated from 125I-human serum albumin measurements (17). Several other studies had revealed an independent correlation between increased levels of PV estimates and increased clinical outcome risk (18–20). Hyponatremia and hypoproteinemia were also predictive factors for recent death in patients with heart failure (21). Our research also confirmed that high ePVs group had higher cardiac deaths and heart failure readmissions and were independent risk factors for them. Serum sodium and albumin concentrations were lower in the MACE group.

The left atrium could be compensated by pressor function in the early stage of heart failure. With the continuous increase of left ventricular end-diastolic pressure, the left atrium will continue to expand and eventually exceed its own regulatory range, and eventually decompensated, leading to the reduction of left atrial function in all aspects (22). Left atrial volume was an independent predictor of cardiovascular events, including atrial fibrillation, heart failure, stroke, and death (23). Left atrial diameter was proved to have a strong correlation with left atrial volume (24). The study also confirmed that the LAD was larger in the MACE group and was an independent risk factor for predicting MACE. Because fibrillation/flutter was closely associated with atrial remodeling, which affected the size of the left atrium. Therefore, atrial fibrillation/flutter was included as an exclusion criterion in our study. Considered the high prevalence of atrial fibrillation/flutter in a HF population aged >60 years-old, the exclusion of patients with atrial fibrillation/flutter may be an important limiting factor to generalize the conclusion of the study to all patients with acute failure.

There were some limitations to the study. Firstly, the major limitation of our study is the population size and the size of the MACE group, which are really small to confidently assess such a clinical endpoint. Secondly, due to the high clinical workload, the index of left atrial diameter was used instead of left atrial volume. Finally, there was no dynamic observation of the patient’s volume profile, which may have had some impact on the results.

## Conclusion

Estimated plasma volume status and LAD have predictive value for assessing the incidence of cardiogenic death and heart failure readmission in acute heart failure patients. It is easy to detect, cheap and has promising utility for the prediction of cardiovascular death and heart failure readmission in patients with AHF.

## Data availability statement

The original contributions presented in this study are included in this article/supplementary material, further inquiries can be directed to the corresponding author.

## Ethics statement

The studies involving human participants were reviewed and approved by Ethical Review Board of the Affiliated Hospital of Putian University. Written informed consent for participation was not required for this study in accordance with the national legislation and the institutional requirements.

## Author contributions

XC and GL designed and produced the manuscript. KX analyzed the data. CD conducted study and reviewed the manuscript. All authors contributed to the final manuscript, read and approved the submitted version.
